# Evidence for novel epigenetic marks within plants

**DOI:** 10.3934/genet.2019.4.70

**Published:** 2019-12-24

**Authors:** Asaad M Mahmood, Jim M Dunwell

**Affiliations:** 1 Department of Biology, College of Education, University of Garmian, Kalar, KRG/Iraq; 2 School of School of Agriculture, Policy and Development, University of Reading, Reading, Berkshire, UK

**Keywords:** epigenetic mechanisms, DNA demethylation, 5hmC, TET proteins

## Abstract

Variation in patterns of gene expression can result from modifications in the genome that occur without a change in the sequence of the DNA; such modifications include methylation of cytosine to generate 5-methylcytosine (5mC) resulting in the generation of heritable epimutation and novel epialleles. This type of non-sequence variation is called epigenetics. The enzymes responsible for generation of such DNA modifications in mammals are named DNA methyltransferases (DNMT) including DNMT1, DNMT2 and DNMT3. The later stages of oxidations to these modifications are catalyzed by Ten Eleven Translocation (TET) proteins, which contain catalytic domains belonging to the 2-oxoglutarate dependent dioxygenase family. In various mammalian cells/tissues including embryonic stem cells, cancer cells and brain tissues, it has been confirmed that these proteins are able to induce the stepwise oxidization of 5-methyl cytosine to 5-hydroxymethylcytosine (5hmC), 5-formylcytosine (5fC), and finally 5-carboxylcytosine (5caC). Each stage from initial methylation until the end of the DNA demethylation process is considered as a specific epigenetic mark that may regulate gene expression. This review discusses controversial evidence for the presence of such oxidative products, particularly 5hmC, in various plant species. Whereas some reports suggest no evidence for enzymatic DNA demethylation, other reports suggest that the presence of oxidative products is followed by the active demethylation and indicate the contribution of possible TET-like proteins in the regulation of gene expression in plants. The review also summarizes the results obtained by expressing the human TET conserved catalytic domain in transgenic plants.

## 1. Introduction

Methylated cytosine (5-methylcytosine, 5mC) a common epigenetic mark in most eukaryotes, is involved in many biological processes that have been extensively documented. It is accepted as the fifth nucleotide base in mammalian and other genomes [1]. Several reviews have discussed the central role of DNA methylation in translational and posttranscriptional gene silencing and subsequently chromatin remodelling [2]. It is indicated that the direction of methylation through the potential role of siRNAs in the nucleus is associated with heterochromatin formation. This process underlies the guiding of the nascent complementary RNA scaffold by siRNA and results in the recruitment of proteins controlling covalent chemical changes on histone tails and DNA methyltransferases [3]. Subsequently, siRNA silences transcription and then mediates modifications in chromatin structure [4]. Furthermore, a significant enrichment of DNA methylation, consistent with previous studies, was found in heterochromatin and siRNA clusters and also has a pivotal role in silencing transposons [5]. Another more recent study explained the direct contribution of DNA methylation to the regulation of endogenous gene expression [6].

It has been widely demonstrated that modification of plant genomes by an array of epigenetic marks is involved in the regulation of plant growth and development [5],[7] and in imprinting, an important phenomenon found in both plants and animals [8]–[11]. Methylation, both of histone tails, particularly on the lysine 4, 9, and 27 positions, and of DNA is an important determinant of epigenetic marks of plants when compared with other types of DNA and histone modifications such as acetylation, phosphorylation and ubiquitination [12]. In addition, the presence of 5mC in the genomes of higher plants is very common and plays several roles including regulation of gene expression that is important in the process of mobilisation and activation of transposable elements in which the high frequency of this epigenetic mark reduces this mobilization. The modified base 5-mC is also important in the regulation of gene expression during development [13]–[15].

Cytosine methylation primarily occurs at CG dinucleotide sites, although CNG and CNN sites can also be significantly methylated in plants [16]. The levels of this epigenetic element and their location differ significantly among plants and animals [17],[18]. For example, despite the relatively low overall levels (approximately 4%) of total cytosine methylation in human genomes, the majority (60–80%) of CG dinucleotides are methylated. The frequency and pattern vary significantly according to cell type and also vary in specific diseases [19]. The frequency in mammals contrasts with plants, where depending on the species, genomes contain higher levels of cytosine methylation in the range from 5–25% [20]. In Arabidopsis, methylation occurs at a frequency of 22.3, 5.92 and 1.51% of CG, CHG, and CHH sites, respectively [17].

This review was undertaken in order to assess the evidence relating to the possible presence of oxidised derivatives of 5mC with plant genomes, and to consider the hypothesis that plants contain equivalents to the TET-like enzymes present in animals.

## 2. Erasers of 5mC

**Figure 1.**
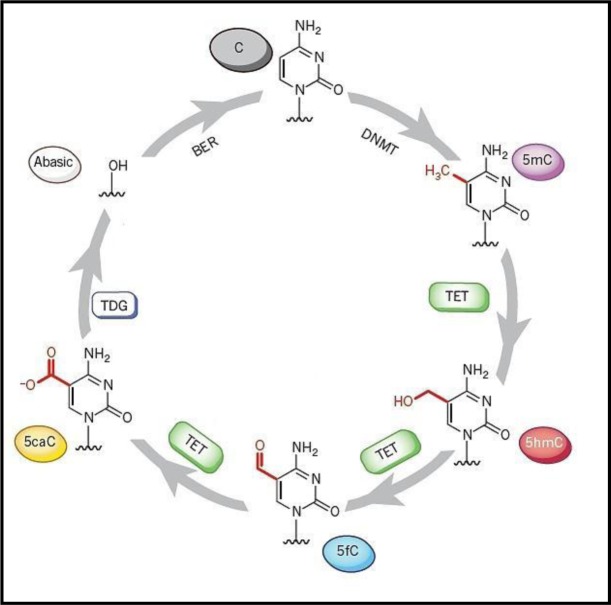
A putative cycling of cytidine derivatives via methylation and oxidative methylation. Where: C = Cytosine, 5mC = Methyl cytosine, 5hmC = 5-Hydroxycytosine, 5fC = 5-Formylcytosine and 5caC = 5-Carboxycytosine, BER = Base Excision Repair, TDG = Thymine DNA glycosylase, DNMT = DNA methyltransferase and TET = Ten-eleven translocation. Modified from [29],[33],[34].

**Figure 2.**
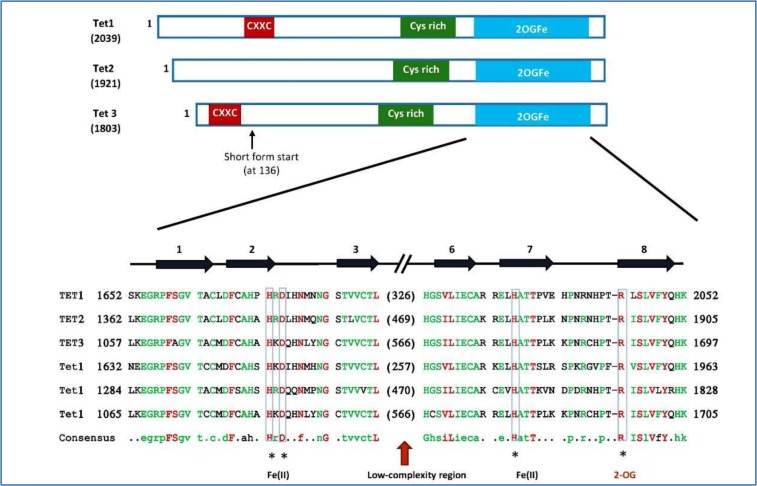
Schematic diagram of the Tet proteins in *Trypanosoma brucei*, human and mouse. The three conserved domains indicated in Tet proteins, include a CXXC zinc finger, a cysteine-rich region (Cys-rich), and a double-stranded β-helix (DSBH) fold of the 2(OG)-dioxygenase domain. Modified from [35].

It has been suggested that the epigenetic information including DNA methylation of the epigenome can be maintained and also be translated through the dynamic activity of DNA methylases (writers), demethylases (erasers) and also reader proteins. Collectively, these proteins are responsible for the recognition and interpretation of the information in both mammals and plants [21]–[23]. Despite its involvement in regulating transcription and developmental growth, 5 mC methylation in plants and animals can be removed passively, such as by DNA replication over subsequent cleavage in the absence of remethylation, or can be actively demethylated through the activities of enzymes. This active removal is established through at least three hypothetical routes, (i) direct removal of the methyl group from the cytosine ring, (ii) the self-excision of methylated cytosine or (iii) chemical modification and remodelling of 5mC residues [24]. Demethylation in mammals [25] and other advanced metazoans can be accomplished by the function of enzymes such as Ten-eleven Translocation (TET) enzymes, which progressively oxidise 5mC to 5hydroxymethy cytosine (5hmC), formyl cytosine (5fC), and then carboxy cytosine (5caC), [26]–[28] (see discussion below). The dynamics of DNA demethylation is the subject of a relevant review [29], which explains that 5fC and/or 5caC are products of TET iterative oxidation that might be subsequently excised via the Base Excision Repair (BER) pathway [30],[31] (Figures 1 and 2). This process, which corrects small base lesions caused by methylation, oxidation or deamination, initially involves a DNA glycolase. This enzyme removes the damaged base and leaves an abasic site, which is later modified by either a short-patch or long-patch repair. This issue, and evidence the possible direct reversion of 5fC to cytosine, have been recently discussed [25],[32].

**Figure 3.**
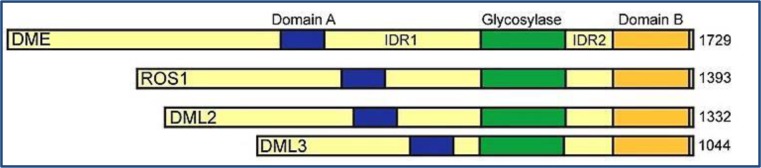
Biochemical representation of the *Arabidopsis thaliana* DME glycosylase domain. Blue, green and orange boxes represent three conserved domains of DNA glycosylases indicating domain A, glycosylase and domain B respectively, modified from [42]. Demeter (DME), Repressor of silencing1 (ROS1), Demeter-like2 (DML2), Demeter-like 3 (DML3), and Intrinsically disordered region (IDR).

In terms of their function, all TET family members have an active role in regulating the transcription of genes [36]. For example, during embryonic stem cell (ESC) differentiation TET proteins and 5hmC are both strongly regulated [37]–[39]. The hypothesis that suggests that 5hmC is produced in ESCs under certain physiological conditions in response to the TET enzymes has been strongly supported [34]. In addition, researchers have identified that the three distinct TET proteins in mice demonstrate different expression patterns [40], although as in all mammals the three proteins share common structural characteristics. These features include a cysteine-rich region and a C-terminal catalytic domain that comprises a double-stranded β-helix (DSBH) or cupin fold [41] with an HXDXnH motif (Figure 3).

It has also been demonstrated that TET1 has a role in maintaining the stem cell state in embryonic stem cells via interaction with the promoter of the *Nanog* gene [43], which then generates a balance of hypermethylation of the promoter [34]. Both TET1 and TET3 have a CXXC DNA binding domain [44], which was identified as a CpG-binding motif, and might promote the recruitment of the above TET members to DNA. It is recently described that mutating a conserved domain site Thr1372 residue of human TET2 is able to make the protein predominantly oxidize 5mC to 5hmC with little to no 5fC or 5caC formed, thereby significantly shifting the substrate preference [45].

## 3. Genetic effects of changes to DNA methylation patterns

The importance of 5hmC and the ubiquitous occurrence of *TET* genes in mammals and other metazoa have been extensively studied [46]. In combination with 5mC, 5hmC plays a significant role in many specific genome functions such as zygotic development in mammals [47] and other species. For example, 5hmC is identified at a high level in promoters as well as in intragenic regions (gene bodies) [48]. Other relevant studies have observed the preferential localization of 5hmC within gene bodies. This modified base is more abundant in exons in comparison with introns [49]–[51]. Overall, 5hmC is considered to be important in the regulation of gene expression [52]–[54] in which its existence is highly correlated with up-regulation of corresponding genes [55],[56]. Furthermore, the functional role of this biomarker was elucidated in development and neuronal activity [57],[58]. In mouse cerebellar Purkinje neurons, abundance of 5hmC is nearly 40% of that of 5mC, whereas spleen and thymus have a low 5hmC level (5–15%). It has also been found 5hmC abundance is negatively correlated with cell proliferation [45]. The importance of this epigenetic modification and its association with the pluripotent state during embryogenesis was first revealed by [37],[39]. An embryogenesis study of DNA demethylation of the mouse zygotic paternal genome demonstrate important implications of active zygotic demethylation in genomic imprinting and X-chromosome inactivation following dramatic changes in DNA methylation after fertilization [59]. Moreover, neural circuit activity linked with DNA modification elucidated the active roles of DNA demethylation in modulating neurogenesis in the adult brain [60]. Moreover, a study of how the development of tumours in human breast, liver, lung, pancreatic and prostate cancers is associated with levels of 5hmC and TET gene expression, has identified a broad and tight association of 5hmC with tumour development [7]. In this latter study, a substantial decrease in the expression of all three *Tet* genes in association with a low level of 5hmC was reported. In summary, all this evidence, together with additional studies has revealed the significant role of 5hmC in embryogenesis [37],[39] and development of mammalian tissues [57],[58]. However, the presence of an active enzymatic demethylation process, the possible enzymes involved, its genome-wide distribution and the possible epigenetic roles of 5hmC in plants are still unclear.

In plants, many studies have been also conducted to investigate the genome-wide distribution of 5mC, its effect on gene expression and its biological function in terms of morphological characteristics and adaptation to unfavourable environment. For example, one study on DNA methylation of *Medicago truncatula* shows the ability of plants to remodel their landscape of DNA 5mC across gene structures under salinity stress [61]. This remodelling varied between gene regions and also between sequence contexts of 5mC in which CG occupied gained a vague impact on the expression levels particular genes in salt tolerant mechanisms. Similarly, it has been observed that a negative correlation exists between gene expression and CG methylation when located within the promoters, while this correlation was positive between gene expression and CNG/CNN methylation when roots of palm were exposed to salinity condition [62].

**Figure 4.**
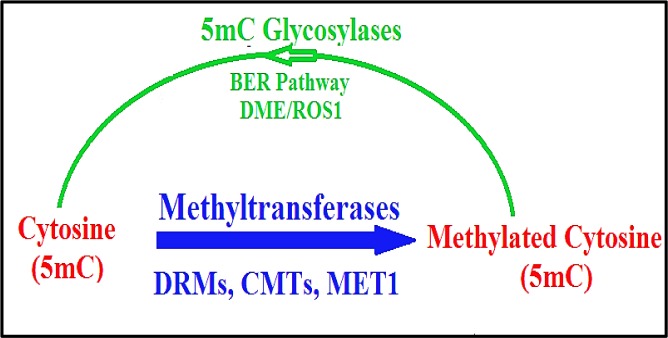
Putative enzymatic DNA demethylation pathway in plants, modified from [67],[68].

As regards the corresponding phenomenon of demethylation in plants, a subfamily of helix-hairpin-helix-Gly/Pro/Asp (HhH-GPD) DNA glycosylases in *Arabidopsis* including DEMETER (DME), REPRESSOR OF SILENCING1 (ROS1), DEMETER-LIKE2 (DML2), and DEMETERLIKE3 (DML3) are involved in active reversion of cytosine methylation [63] (Figure 4).

It has been stated that DME is essential during seed development as a consequence of its involvement in DNA demethylation. It is found to be required in one of the female gametes in regulating active reversion of DNA methylation before fertilization [64]. Two different related studies have found that during vegetative developments, other 5mC DNA glycosylases (DML2, DML3 and ROS1) function to preserve the accumulation of DNA methylation in order to protect genes from potentially deleterious methylation [65],[66].

In contrast to the situation in metazoa, there is limited evidence related to the presence of 5hmC in plants; this has resulted in a controversial debate about the definitive existence of 5hmC as well as functional counterparts of TET-like enzymes [69],[70] (see next section).

## 4. TET enzymes: Are they present in plants?

TET enzymes are members of the oxoglutarate/iron dependent dioxygenases (2-OG)-dioxygenases, which are one of the most functionally diverse super-families of non-haem enzymes. They are distributed in a wide range of prokaryotes and eukaryotes, and are involved in many different important biological activities including biosynthesis of plant products and antibiotics, posttranslational modification, DNA/RNA damage repair and metabolism of lipids [71].

For example, within plants one sub-class of these enzymes comprise anthocyanidin synthase, flavanone 3β-hydroxylase and flavonol synthase [72],[73]. These particular enzymes are responsible for biosynthesis of flavonoids, secondary products, which are ubiquitous in spermatophytic plants, and fulfil a multitude of physiological roles [74]. These roles include adaptation to biotic and abiotic stresses in which flavonoids function in helping plants to cope with stress by modulating fertility and regulating the transport of auxin, an important plant hormone [75]. Synthesis of another class of plant hormones, namely gibberellic acids (GAs), involve the activity of a related group of 2(OG)-dioxygenases, which include GA 20-oxidase (GA20ox) and GA 3-oxidase (GA3ox) [72]. Loss of expression of *AtGA20ox1* in an *Arabidopsis* mutant reduced internode length and subsequently stem height [76], while similar traits in rice (*Oryza sativa*) and barley (*Hordeum vulgare*) containing mutations (eg. Semi-dwarf, *sd1*) in the cereal GA20ox2, have been selected by breeders for lodging resistance and subsequently increased yield [77],[78].

The presence of more than 130 dioxygenase genes has been reported in the genome of *A. thaliana*. This superfamily of dioxygenases includes a group of important catalytic enzymes named Prolyl 4-hydroxylases (P4H) that are found in mammals and plants [79]. This group of enzymes in mammals has been well described and divided into two types according to their localization; first, the collagen-type-P4H that are localized in the endoplasmic reticulum and secondly hypoxia-inducible factor (HIF)-P4Hs that are localized in the cytosol [80],[81]. These enzymes are involved in regulating epigenetic processes, metabolic reactions and possible therapeutic use in mammals [82]. Similarly, a large group of homologous genes encoding such enzymes are present in plants; for example, thirteen putative P4H genes, numbered from *ATP4H1*-*ATP4H13*, have been described in *Arabidopsis* [83]. The diversity of substrate and the large number of related enzymes with no known function provide the possibility of plant 2(OG)-dioxygenases (TET-like proteins) responsible for the regulation of epigenetic processes via active DNA demethylation. Of course, such enzymes would need to be part of a complex that included protein(s) with a DNA binding motif equivalent to the CXXC domain of the TET enzymes [44].

## 5. Oxidative derivatives of 5mC in plants

Previous studies confirmed that glycosylases can directly remove 5mC from the DNA backbone and subsequently might create an abasic site as a part of the BER pathway [68]. However, global demethylation was not determined when known demethylases in *Arabidopsis* are mutated; the methylation status was only affected at some specific loci [64],[66]. Moreover, due to the generation of several abasic sites which might simultaneously break strands, the BER pathway might not be the main process for global demethylation leading to destabilize the whole genome [84],[85]. However, it is still not possible definitely to confirm the occurrence of global demethylation during the reproductive processes of Table gametogenesis and embryogenesis [85]. By comparison with the demethylation pathway by TET enzymes in mammals, is there an equivalent demethylation pathway in plants?

To determine whether oxidation products of 5mC are present in plant DNA, different methods with various sensitivities and specifications have been used. A series of studies [86]–[88] used dot-blot assays and the liquid chromatography-multi-stage mass spectrometry (LC-MS/MS/MS) method to identify the presence of 5hmC as shown in (Table 1).

**Table 1.** Content of 5hmC in (A) genomic DNA and (B) RNA in different tissues of various plants using several methods.

Sample	5-hmC as % of total cytosine	Method	Comment	Reference
(A)				
Leaves, flowers of *A. thaliana*	0.055	Fluorescent antibody	Suggested further studies with advanced techniques	[89]
Leaves, flowers of *A. thaliana*	0.068–0.075	Antibody-based dot-blot	Suggested passive DNA demethylation	[86]
Protoplasts of *Cucumis sativus*, *Brassica oleracea*	0.2–0.3	Fluorescent antibody	Removal of DNA methylation may result from biological activity rather than oxidative DNA damage	[87]
Leaves of *A. thaliana*	0.033	Liquid chromatography-multi-stage mass spectrometry (LC-MS/MS/MS)	Observed passive DNA demethylation as a result of oxidative DNA damage, and suggested further studies to detect other oxidative compounds, foC and caC	[88]
Leaves, floral buds of *A. thaliana*	Undetectable in comparison with 0.2% synthetic DNA with 5hmC	Thin layer chromatography (TLC)	No 5hmC present as an intermediate of DNA demethylation	[69]
Leaves, panicles of *Oryza sativa L. ssp. indica*	1.39 and 2.17/10^6^	LC-MS/MS/MS	Detected higher levels of 5hmC in heterochromatin regions, particularly in transposable elements (TE)-related genes. Suggest significant association between chromatin structure and levels of 5hmC.	[90]
Shoots of *A. thaliana*; Flower bud of *A. thaliana*; Seedling, shoot, root, leaves of *A. thaliana and Z. mays*; Seeds, pollen of *A. thaliana*	Range from 0.07–0.17. Higher level in seedling of both species.	TLC; Antibody; Enzyme-linked immunosorbent assay (ELISA); β-glucosyltransferase radiolabeling MS	Not detectable, 5hmC less than control of 0.5%; Detectable but less than control; Quantity not biologically significant; Not detectable	[91]
Leaves of heat-stressed Brassica napus	0.324	Methyl flash hydroxylated DNA quantification (colormetric)	Slight increase in level of 5hmC in line with increase of 5mC in response to heat stress	[92]
Leaves of a range of *O. sativa* cultivars	1.32/10^6^ to 1.98/10^6^	Chromatography-tandem mass spectrometry with isotope dilution	Levels of 5hmC lower than in animals	[93]

(B)				
*A.thaliana*	0.00485	LC-MS/MS	First report of 5hmC in plant RNA	[94]

The detection of 5hmC has been reported in leaves and flowers of *Arabidopsis* at a frequency 0.07% of total cytosines, suggesting that this derivative results from passive loss of DNA methylation following DNA replication [86]. In support of this idea, other studies have observed that the iterative oxidation of 5mC has been found at a low level comparable to the lowest level in mammals (0.15%–0.3%), and suggested that the 5hmC detected has been generated from non-enzymatic DNA demethylation [69],[88],[91]. On the other hand, it has been suggested that the presence of 5hmC is an oxidative product of 5mC followed by the active demethylation, rather than being the result of oxidative DNA damage [87],[90].

Moreover, significant enrichment of 5hmC in heterochromatin regions, particularly in transposable element (TE)-related genes, has been identified in rice. This result led researchers to suggest a significant association between chromatin structure and levels of 5hmC [90]. Although some observations claimed that the presence of 5hmC resulted from a passive reversion of DNA methylation in plants, it was also suggested further studies are needed definitively to detect other oxidative compounds including 5fC and 5caC.

In line with this suggestion, one comprehensive study used chemical derivatization coupled with liquid chromatography/Tandem mass spectrometry analysis to determine oxidative products of 5mC in samples from plants including *A. thaliana*, *Lycopersicon esculentum, Ginkgo biloba, Platycladus orientalis, Zea mays*, and *Oryza sativa* [95]. Results from this study identified the widespread presence of further oxidative products, including 5fC and 5caC, from genomic DNA of several different tissues. Apart from the possible direct cleavage involving the DNA glycosylase pathway in combination with the BER pathway, these results led the authors to conclude that in a similar way to the DNA demethylation pathway in mammals, there is an alternative active reversion of DNA methylation in plants as well. Furthermore, they observed different levels of 5fC and 5caC in plant genomes under drought and salinity stress conditions, suggesting biological functions of both epigenetic elements under environmental stress conditions. Recently, LC-MS/MS analysis was used to study the abundance of 5hmC in RNA of plants including *Arabidopsis thaliana* [94]. These authors identified the presence of 5hmC in RNA, and suggested that this 5hmC results from active (enzymatic) transformations. Moreover, they suggested that oxidation products of 5mC may have critical regulatory functions.

Although oxidative products of 5mC have been detected in plants, as previously mentioned, there is no evidence to confirm the involvement of plant enzymes equivalent to the TET family proteins as described in mammals. Again as mentioned previously, there is evidence for some functional redundancy for *Tet1, 2, 3* genes in mammals [96]. It is generally assumed [97],[98] that these three genes are not present in multicellular plants, despite the fact that in many ways the plant kingdom is as diverse and complex as that of the metazoans and fungi. The logical corollary to the assumption of the absence of *Tet* genes is that there are no oxidised products of 5mC. However, previous data on the presence of oxidative derivatives of 5mC led to the conclusion that there must be another family of protein(s) responsible for the production of DNA modifications and its biochemistry may be similar to that of the TET protein family because of the presence of the intermediate and terminal oxidation products such as 5hmC (see section below).

## 6. Expression of human TET domains in transgenic plants

After the discovery of the functional role of human TET proteins it became of immediate interest to examine the possible activity of such proteins when expressed in plants. The first such published study was that in which the C-terminal catalytic domain of the human Tet3 gene, under the control of the constitutive 35S promoter, was transformed into *Arabidopsis* [99]. A rDNA region was used as a methylation reporter and it was found that epialleles with either a hypomethylation or hyper methylation pattern could be induced. These patterns were stably retained even after removal of the transgene. In these TET3 transformants 5 hmC marks were detected; this was indicative of the oxidative capacity of the transgenic enzyme. In addition, 5 fC was only detectable in transformants with a DNA glycosylase background, a finding that suggests further oxidation of 5 hmC residues to 5fC by the human catalytic domain. Using the same construct, the same group extended this work to tomato where they generated transformants with distinct phenotypes, which included plants with an increase in the length and number of leaves [100]. The authors identified in these transformants various changes in the expression of CEN1.1, a member of the phosphatidyl ethanolamine-binding protein (PEBP)/centroradialis, terminal flower1, self-pruning (CETS) family. These changes were linked to hypomethylation of the gene and its consequent activation in leaves.

Three other groups have also reported results from similar [101] and more complex studies [102],[103]. In the former study, the catalytic domain of the human TET1 protein, again under the control of the 35S promoter, was introduced into Arabidopsis. Analysis of two transformants revealed global reduction of CG methylation, from 18.2% in the wild-type to 8.9% and 6.9% respectively. There were smaller effects on CHG and CHH methylation. In contrast to the previous reports [99], the authors of the second study [101] found no evidence of 5hmC in their transformants. They concluded that this absence of 5hmC may be linked to the reduction of CG methylation, or through the active removal of 5hmC or further oxidised products via the BER pathway. A possible, though more unlikely explanation for the differences in 5hmC levels between the two studies on Arabidopsis may lie in differences between the activities of the TET1 [102] and TET3 [99] catalytic domains. In this context, it is known from studies of the human TET3 isoforms that they have different patterns of expression and substrate binding [44].

The second group who expressed human TET domains in plants used a construct in which a section of the Arabidopsis ubiquitin10 promoter mediated expression of the human TET1 catalytic domain (TETcd) fused to an artificial zinc finger (ZF) designed to target the promoter of the *FLOWERING WAGENINGEN* (*FWA*) gene in Arabidopsis [102]. The loss of cytosine methylation in the promoter of this gene is known to promote a heritable late-flowering phenotype and such a phenotype was found in the TET1 transgenics. The authors of this study also developed a ZF-TETcd fusion to target methylated regions of the *CACT1* transposon and thereby generated targeted demethylation. In addition, they developed a CRISPR/dCAS9-based targeted demethylation [104].

The third group who used this approach generated a plasmid containing the catalytically inactive SpCas9 joined to human TET3cd (aa 850–1795) [103]. They used a derivative of this plasmid and four guide RNAs to target a specific gene from *Brassica oleracea* shown to be highly methylated in the progeny of a microspore-derived doubled haploid [105]. The resultant plants showed reduced methylation and a noticeable increase in expression of this targeted gene.

In combination, these TET-based molecular tools demonstrate the possibility of generating new epialleles of genes of interest, as well as reactivating expression of silenced genes, transgenes or transposons [106].

Taken together, these four studies provide definitive evidence that the human TET catalytic domains are active when expressed in plants and they may be capable of generating additional epigenetic marks by oxidation of 5mC and thereby producing stably inherited phenotypic variation.

## 7. How to identify TET homologues in plants

The present absence of direct functional evidence for TET-like enzyme activity in plants, as discussed above, raises the question about how a search for such evidence should be best conducted. There are a number of options, which include the following. First, all the plant 2-OG oxygenases with known or unknown substrate(s) could be overexpressed in a heterologous system, purified, and then tested on a range of synthetic DNA and RNA [107],[108] with various 5mC contexts in a search for the known 5mC derivatives using the latest, high-resolution analytical [109]–[115] and bioinformatic methods [116] methods. Secondly, Arabidopsis or other plant mutants in all known 2-OG oxygenases could be similarly investigated for the presence of 5mC derivatives. In this case, it might be necessary to conduct detailed single cell [117], or other microscale techniques, in order to take account of the possibility that expression of TET-like genes is limited to specific cell types, as is the situation in humans [48],[118] and other metazoans, for example Drosophila [119],[120].

A second question raised by any search for TET-like enzymes is whether the oxidative function of these enzymes might be affected by evolutionarily non-related enzymes. Such enzymes would fall under the definition being non-homologous (analogous), isofunctional proteins [121]. One search for examples of such proteins identified 185 Enzyme Commission (EC) numbers that included two or more proteins without detectable mutual sequence similarity [122]. In terms of function these enzymes include cellulases [123] and some involved in methionine biosynthesis [124]. In other words, it is theoretically possible that the oxidation of 5mC and its derivatives might be accomplished by oxidases other than the 2-OG oxidases.

## 8. Conclusions

Despite the extensive experimental evidence outlined above, several important questions regarding the biological function of Tet-like proteins in plants remain unanswered. Are there any TET equivalents in plants? To what extent are dioxygenase proteins containing conserved domains similar to those of TETs involved in oxidation of 5mC in plants? This remains a controversial issue that has divided scientists into two main groups. The first group strongly supports the conclusion that there is no biologically relevant 5hmC in plants and particularly *Arabidopsis*. The second group accepts the evidence for the presence of 5mC derivatives generated from active demethylation in a process similar to that in the mammalian pathway. Hopefully, the increasingly sophisticated analytical and molecular techniques will be able to resolve this important issue.
